# Radiotherapy with continued EGFR‐TKIs for oligoprogressive disease in EGFR‐mutated non‐small cell lung cancer: A real‐world study

**DOI:** 10.1002/cam4.4894

**Published:** 2022-06-05

**Authors:** Chunhong Hu, Sixuan Wu, Renfang Deng, Yuanqiang Wu, Yue Pan, Long Shu, Fang Wu

**Affiliations:** ^1^ Department of Oncology The Second Xiangya Hospital, Central South University Changsha Hunan China; ^2^ Department of Internal Medicine Oncology The First Affiliated Hospital of University of South China Hengyang Hunan Province China; ^3^ Department of Oncology ZhuZhou Second Hospital ZhuZhou Hunan China; ^4^ Hunan Cancer Mega‐Data Intelligent Application and Engineering Research Centre Changsha Hunan China; ^5^ Hunan Key Laboratory of Tumor Models and Individualized Medicine The Second Xiangya Hospital, Central South University Changsha Hunan China; ^6^ Hunan Key Laboratory of Early Diagnosis and Precision Therapy in Lung Cancer The Second Xiangya Hospital, Central South University Changsha Hunan China

**Keywords:** EGFR‐mutated non‐small‐cell lung cancer, oligoprogression, radiotherapy, survival, tyrosine kinase inhibitors

## Abstract

**Background:**

Epidermal growth factor receptor (EGFR)‐mutated non‐small cell lung cancer (NSCLC) develops resistance to tyrosine kinase inhibitors (TKIs). Here, we evaluated the efficacy of radiotherapy and continuation of TKIs in patients with advanced NSCLC with oligoprogression after EGFR‐TKIs.

**Methods:**

From January 2011 to January 2019, 33 patients with EGFR‐mutated NSCLC on TKIs were treated by radiotherapy and continuation of TKIs for oligoprogressive disease. The primary endpoints were median progression‐free survival 1 (mPFS1), mPFS2, and median overall survival (mOS). PFS1 was measured from the start of EGFR‐TKIs therapy to the oligoprogression of the disease. PFS2 was measured from the date of oligoprogression to the further progression of the disease, while OS was calculated from oligoprogression to death from any cause or was censored at the last follow‐up date.

**Result:**

The mPFS1, mPFS2, and mOS were 11.0 (95% CI, 4.4–17.6), 6.5 (95% CI, 1.4–11.6) and 21.8 (95% CI, 14.8–28.8) months, respectively. Univariate analysis showed that EGFR mutation type (*p* = 0.024), radiotherapy method (*p* = 0.001), and performance status (*p* = 0.017) were significantly correlated with PFS2. Univariate analysis showed that sex (*p* = 0.038), smoking history (*p* = 0.031), EGFR mutation type (*p* = 0.012), and radiotherapy method (*p* = 0.009) were significantly correlated with OS. Multivariate analysis suggested that radiotherapy method (*p* = 0.001) and performance status (*p* = 0.048) were prognostic factors for PFS2, and radiotherapy method (*p* = 0.040) was a prognostic factor for OS.

**Conclusion:**

Radiotherapy with continued TKIs is effective for EGFR‐mutated NSCLC with oligoprogression, and it should be conducted as soon as possible. T790M+ patients have higher sensitivity to radiotherapy, and patients with good performance status and stereotactic body radiation therapy have better PFS2 and OS.

## Abstract 2

Almost all EGFR mutation‐positive advanced NSCLC patients treated with TKI will progress. The definition of oligometastasis disease included ≤5 metastases with ≤3 organs involved, and all metastatic sites could be amenable to local radical treatment. Radiotherapy with continued TKIs is effective for EGFR‐mutated NSCLC with oligoprogression.

## 1 INTRODUCTION

Non‐small cell lung cancer (NSCLC) is the most common type of lung cancer, accounting for approximately 85% of all cases.^1^ One‐third of patients with NSCLC showed advanced metastatic disease at the time of diagnosis. Somatic driver oncogene mutations such as epidermal growth factor receptor (EGFR) and anaplastic lymphoma kinase (ALK) have been frequently identified in NSCLC.^2^ In the Asian population, 30%–40% of NSCLC patients with adenocarcinoma exhibit EGFR mutation.^3^ EGFR mutation‐positive advanced NSCLC and EGFR tyrosine kinase inhibitors (TKIs) treatment is associated with better survival outcomes and fewer side effects than treatment with standard first‐line platinum‐based chemotherapy.^4^, ^5^ However, almost all patients will progress, with a median progression‐free survival (PFS) of 10–14 months and a median overall survival (OS) of 38 months.

Oligometastatic disease was first described by Hellman and Weichselbaum in 1995 as a status between metastatic systemic disease and localized disease.^6^ The definition of oligometastatic disease varies, but most cases indicate fewer than five sites of metastasis. Recently, a new definition was proposed by a European expert group that included ≤5 metastases with ≤3 organs involved, and all metastatic sites could be amenable to local radical treatment.^7^ Oligoprogressive sites are resistant to TKIs, however, some tumor sites might still be sensitive to TKIs, and continuation of EGFR‐TKIs may provide additional benefits in patients with oligoprogression. Radiotherapy to oligoprogressive sites might restore the sensitivity of the metastatic disease and improve the survival outcome.^8^, ^9^ A retrospective matched cohort study investigated the clinical outcome of EGFR mutation‐positive stage IV NSCLC receiving radiotherapy or chemotherapy for progression and the results showed that the mPFS was significantly higher in radiotherapy group than chemotherapy group (7.0 vs. 4.1, *p* = 0.0017), indicating that radiotherapy could extend EGFR TKI therapy for patients with oligoprogression.^10^ There is limited evidence for oligometastatic radiotherapy after EGFR mutation‐positive NSCLC targeted therapy, but it is unclear what kind of patients are the dominant population and the time of oligoprogression, and whether radiotherapy technology has an effect on efficacy.

## 2 METHODS

### 2.1 Patient selection

We conducted a retrospective analysis of NSCLC patients who developed oligoprogression during TKIs therapy between January 2011 and January 2018 at the Second Xiangya Hospital of Central South University. The study participants were registered according to the following inclusion criteria: (i) histological or cytological confirmation of advanced NSCLC; (ii) presence of tumor that harbors an EGFR mutation known to benefit from treatment with EGFR‐TKIs and developed oligoprogressive disease during EGFR‐TKIs therapy; (iii) treatment with continued EGFR‐TKIs therapy and radiotherapy for oligoprogressive disease until further progression (radiotherapy was administered to 33 tumor sites in the brain, lungs, and bones); (iv) absence of severe comorbid conditions; (v) willingness to provide written informed consent. This study was performed in accordance with the Declaration of Helsinki and approved by the Institutional Review Board of the National Cancer Institute and the Ethics Committees of the Second Xiangya Hospital (Ethical approval number: 202008330).

### 2.2 Treatment methods

After determining oligoprogression, all patients were treated with radiotherapy for oligoprogressive lesions. The sites, methods, and dosages of radiotherapy were as follows. Brain: (i) Whole‐brain radiotherapy at 40 Gy/20 F or 30 Gy/10 F; (ii) 6MV‐X line intensity‐modulated radiation therapy (IMRT) at 54–60 Gy/27–30 F; (iii) stereotactic radiosurgery at 30–40 Gy/8–10 F. Lung: (i) IMRT at 50–60 Gy/25–30F; (ii) stereotactic body radiation therapy (SBRT) at 50–60 Gy/5–10 F. Bone: General radiotherapy, 40 Gy/20 F. EGFR‐TKIs were continued in combination with loco‐regional radiotherapy to the site of oligoprogression until further progression. The EGFR‐TKIs included gefitinib (250 mg, once a day), erlotinib (150 mg, once a day), and icotinib (125 mg, three times a day) in our study. The date of progression was defined based on routine surveillance imaging and/or symptomatic progression that prompted earlier radiographic evaluation with routine imaging every 2–3 months for most patients.

### 2.3 Follow‐up and evaluation of the treatment response

The date of progression was defined based on routine surveillance imaging, which prompted earlier radiographic evaluation with routine imaging every 2 months for most patients. Routine surveillance imaging included chest and abdominal computed tomography. Patients underwent bone scan or brain magnetic resonance imaging when bone or brain metastasis was suspected, and positron emission tomography scan was performed when systemic progression was suspected. Treatment response was evaluated in accordance with the Response Evaluation Criteria in Solid Tumors (RECIST) ver. 1.1.

### 2.4 Statistical analysis

The primary research points were the median progression‐free survival 1 (mPFS1), mPFS2, and median overall survival (mOS). PFS1 was measured from the start of front‐line EGFR‐TKIs therapy until the oligoprogression of the disease. PFS2 was measured from the date of oligoprogression until further progression of the disease. OS was calculated from oligoprogression to death from any cause or was censored at the last follow‐up date. The prognostic roles of the clinical and pathologic variables were tested. The prognostic factors selected for analysis included sex (male vs. female), age (>61 years vs. ≤61 years), smoking status, performance status (PS) score (0 to 1 vs. 2), initial resectable (resectable vs. unresectable), radiotherapy method (SBRT vs. non‐SBRT), EGFR mutation type (exon 19 deletion vs. exon 21 L858R mutation), number of metastatic sites (1 vs. >1), sites of radiation (brain vs. lung vs. bone), T790M status, and time of oligometastasis to radiotherapy (>1 month vs. ≤1 month). Univariate analysis was performed to analyze the possible prognostic factors. Selected factors (*p* < 0.10) were evaluated using multivariate analysis with the Cox regression model. In the multivariate analysis, *p* < 0.05 was considered statistically significant.

## 3 RESULTS

### 3.1 Patient characteristics

The patient characteristics are listed in Table 1. In this study, 18 (54.5%) patients were female, and the median age was 61 years (range, 39–82 years). In addition, 26 (75.8%) patients had a PS score of 0 to 1, 26 (78.8%) were initially unresectable, and 20 (60.6%) were never smokers. In terms of EGFR mutations, 17 (51.5%) patients had exon 19 mutations, and 16 (48.5%) had exon 21 mutations. Moreover, 12 (36.4%) patients had radiotherapy for brain metastases, 14 (42.4%) had radiotherapy for lung metastases, and 7 (21.2%) received radiotherapy for bone metastases after TKIs resistance. Nine patients were also treated with SBRT. The time from oligoprogression to radiotherapy was ≤1 month in 19 (57.6%) patients and >1 month in 14 (42.4%) patients. The number of oligoprogression sites was 1 in 17 (51.5%) patients and >1 in 16 (48.5%) patients. The T790M mutation was tested in nine patients: Five patients were positive and four patients were negative.

**TABLE 1:** Patients characteristics

Variable	Patients (*n* = 33)	*N* (%)
Gender		
Male	15	45.5
Female	18	54.5
Age		
Median age (range)	61 (39–82)	
Performance status score		
0–1	25	75.8
2	8	24.2
Smoking status		
Non‐smoker	20	60.6
Smoker	13	39.4
Initial resectable		
Resectable	7	21.2
Unresectable	26	78.8
Radiotherapy methods		
SBRT	9	27.3
Not SBRT	24	72.7
Number of metastasis		
1	17	51.5
>1	16	48.5
EGFR mutation		
Exon 19	17	51.5
Exon 21	16	48.5
Sites of radiation		
Brain	12	36.4
Lung	14	42.4
Bone	7	21.2
T790M status		
Positive	5	15.2
Negative	4	12.1
Time from first PD to radiotherapy		
>1 month	14	42.4
≤1 month	19	57.6

Abbreviations: EGFR, epidermal growth factor receptor; PD, progressive disease; SBRT, stereotactic body radiation therapy.

### 3.2 Survival and prognostic analyses

**FIGURE 1:**
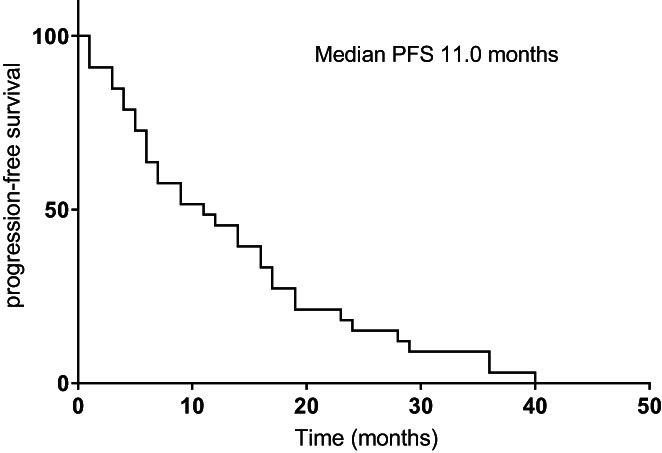
The Kaplan–Meier curve for progression free survival of resistance to EGFR‐TKIs is shown for the 33 patients. The median progression‐free survival was 11.0 months.

**FIGURE 2:**
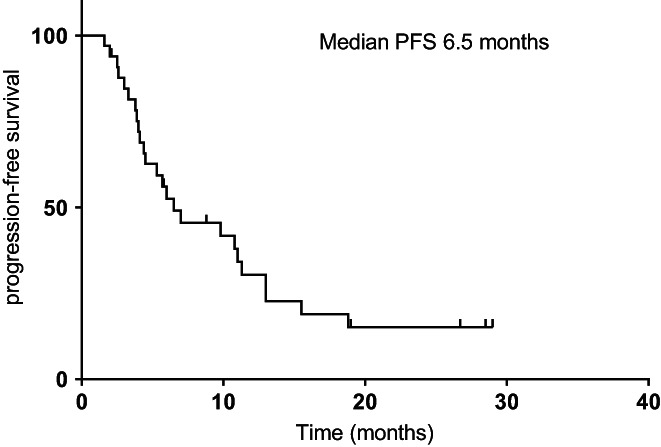
The Kaplan–Meier curve for progression‐free survival from the time of oligoprogression is shown for the 33 patients. The median progression free survival was 6.5 months.

**FIGURE 3:**
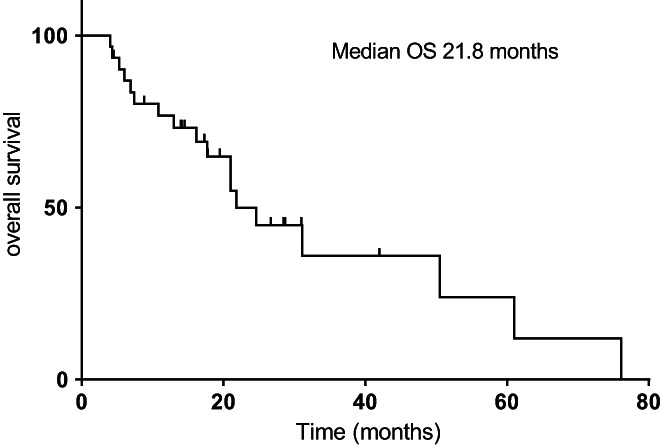
The Kaplan–Meier curve for overall survival from the time of oligoprogression is shown for the 33 patients. The median overall survival was 21.8 months.

**FIGURE 4:**
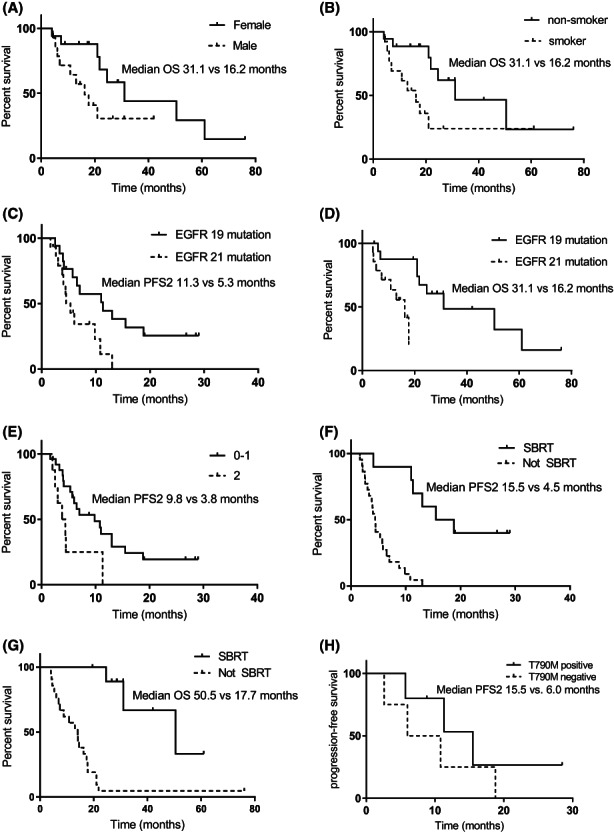
The effect of different clinical factors on survival. mPFS2: the median progression‐free survival after oligoprogression; mOS, the median overall survival after oligoprogression; EGFR. epidermal growth factor receptor; SBRT, stereotactic body radiation therapy. (A). The mOS was 31.1 months in female patients and 16.2 months in male patients (HR = 0.319, *p* = 0.038); (B). The mOS was 31.1 months in non‐smokers and 16.2 months in smokers (HR = 2.963, *p* = 0.031); (C). The mPFS2 was 11.3 months in patients with EGFR exon 19 deletion and 5.3 months in patients with EGFR exon 21 L858R (HR = 2.755, *p* = 0.024); (D). The mOS was 31.1 months in patients with EGFR exon 19 deletion and 16.2 months in patients with EGFR exon 21 L858R (HR = 7.814, *p* = 0.012); (E). The mPFS2 was 9.8 months in patients with a PS score of 0 to 1 and 3.8 months in with a PS score of 2 (HR = 3.121, *p* = 0.017); (F). The mPFS2 was 15.5 months in Patients with SBRT treatment and 4.5 months in Patients without SBRT treatment (HR = 8.203, *p* = 0.001); (G). The mOS was 50.5 months in Patients with SBRT treatment and 17.7 months in Patients without SBRT treatment (HR = 4.947, *p* = 0.009); (H). The mPFS2 was 15.5 months in T790M mutation positive patients and 6.0 months in negative patients (HR = 0.471, *p* = 0.33).

The median follow‐up time was 18 months. The mPFS1, mPFS2, and mOS were 11.0 (95% CI, 4.4–17.6), 6.5 (95% CI, 1.4–11.6), and 21.8 (95% CI, 14.8–28.8) months for the whole cohort, respectively (Figures 1, 2, 3). Univariate analysis revealed that male patients had significantly shorter mOS than female patients (HR = 0.319, *p = 0.038*) (Figure 4A). Never‐smokers also had significantly longer mOS than former/current smokers (HR = 2.963, *p* = 0.031) (Figure 4B). Patients with EGFR exon 19 deletion had significantly better mPFS2 and mOS than those with EGFR exon 21 L858R mutation (mPFS2: HR = 2.755, *p = 0.024*; mOS: HR = 7.814, *p = 0.012*) (Figure 4C,D). Patients with a PS score of 0 to 1 had significantly better mPFS2 scores than those with a PS score of 2 (HR = 3.121, *p =* 0.017) (Figure 4E). Patients who underwent SBRT treatment had significantly better mPFS2 and mOS than those who did not (mPFS2: HR = 8.203, *p = 0.001*; mOS: HR = 4.947, *p =* 0.009) (Figure 4F,G). The mPFS2 was 15.5 months in T790M+ patients and 6.0 in T790M− patients (*p* = 0.330), and mOS was 76.1 months and 31.1 months (*p* = 0.344), respectively (Figure 4H). Patients with one or more than one metastatic site showed an mPFS2 of 10.8 months and 4.4 months, respectively (*p* = 0.218), and an mOS of 24.6 months and 21.0 months, respectively (*p* = 0.340). The mPFS2 in patients who started radiotherapy within or beyond 1 month after oligometastasis was 10.8 months and 5.3 months, respectively (*p* = 0.576), and the mOS was 24.6 months and 21.0 months, respectively (*p* = 0.635). The mPFS2 of patients with postoperative recurrence and initial unresectable was 13.0 and 6.0 months, respectively (*p* = 0.210), and the mOS was 21.0 months and 24.6 months, respectively (*p* = 0.950). Furthermore, the univariate analysis showed that EGFR mutation type (*p* = 0.024), radiotherapy methods (*p* = 0.001), and performance status (*p* = 0.017) were significant prognostic factors for PFS2.

Univariate analysis showed that sex (*p* = 0.038), smoking history (*p* = 0.031), EGFR mutation type (*p* = 0.012), and radiotherapy methods (*p* = 0.009) were significantly associated with OS (Table 2). Multivariate analysis suggested that radiotherapy methods (*p* = 0.001) and performance status (*p* = 0.048) were prognostic factors for PFS2, and that radiotherapy method (*p* = 0.040) was a prognostic factor for OS (Table 3).

**TABLE 2:** Univariable analysis of clinical factors potentially associated with PFS and OS

Variable	PFS1		PFS2		OS	
	HR (95% CI)	*p*	HR (95% CI)	*p*	HR (95% CI)	*p*
Gender	0.886 (0.44–1.79)	0.737	1.085 (0.49–2.40)	0.840	0.319 (0.12–1.13)	0.038
Age (year)	0.756 (0.38–1.52	0.432	1.172 (0.53–2.61)	0.699	1.267 (0.48–3.34)	0.632
Smoking status	0.929 (0.45–1.91)	0.841	1.322 (0.60–2.92)	0.488	2.963 (1.10–7.97)	0.031
EGFR mutation	0.766 (0.37–1.58)	0.472	2.755 (1.14–6.66)	0.024	7.814 (1.57–38.85)	0.012
ECOG performance status	1.497 (0.63–3.55)	0.359	3.121 (1.22–7.98)	0.017	2.229 (0.69–7.24)	0.183
T790M mutation	0.390 (0.09–1.77)	0.222	0.471 (0.10–2.15)	0.330	0.305 (0.03–3.57)	0.344
Time from first PD to radiotherapy	0.667 (0.33–1.36)	0.267	1.251 (0.57–2.75)	0.576	1.263 (0.48–3.30)	0.635
Metastases number	0.879 (0.44–1.77)	0.718	1.646 (0.75–3.64)	0.218	1.608 (0.61–4.27)	0.340
Initial resectable	0.512 (0.22–1.21)	0.125	0.530 (0.20–1.43)	0.210	1.034 (0.36–2.97)	0.950
Radiotherapy methods	0.747 (0.34–1.64)	0.467	8.203 (2.50–26.97)	0.001	4.95 (1.48–16.52)	0.009
Sites of radiation	1.107 (0.69–1.79)	0.677	0.674 (0.37–1.24)	0.203	1.031 (0.49–2.19)	0.936

Abbreviations: ECOG, Eastern Cooperative Oncology Group; HR, hazard ratio; EGFR, epidermal growth factor receptor; OS, the median overall survival after oligoprogression; PD, progressive disease; PFS1, the median progression‐free survival from the start of front‐line EGFR‐TKIs therapy until oligoprogression of disease; PFS2, the median progression‐free survival after oligoprogression.

**TABLE 3:** Multivariable analysis of clinical factors potentially associated with PFS and OS

Variable	PFS2		OS	
	HR (95% CI)	*p*	HR (95% CI)	*p*
Gender	/	/	0.863 (0.16–4.77)	0.865
Smoking status	/	/	2.912 (0.58–14.60)	0.194
EGFR mutation	0.628 (0.22–1.80)	0.386	3.007 (0.57–15.87)	0.195
ECOG performance status	2.963 (1.01–8.72)	0.048	/	/
Radiotherapy methods	12.54 (2.76–57.04)	0.001	4.611 (1.07–19.81)	0.040

Abbreviations: ECOG, Eastern Cooperative Oncology Group; EGFR, epidermal growth factor receptor; HR, hazard ratio; OS, the median overall survival after oligoprogression; PFS2, the median progression‐free survival after oligoprogression.

### 3.3 Toxicity

The toxicities observed, including skin rash, neutropenia, fatigue, anorexia, nausea, and vomiting, were controllable. No G3 to 4 toxicities were recorded, and no other severe acute or late toxicities related to this study treatment were observed.

## 4 DISCUSSION

In the past decade, target therapy such as the EGFR tyrosine kinase inhibitors (TKIs) treatment has significantly improved the survival out advanced‐stage NSCLC with driver oncogene mutations; however, almost all patients develop resistance.^4^, ^5^ The therapeutic strategy beyond progression needs to be determined according to the progression patterns.^11^ For the oligoprogressive disease, discontinuation of TKIs may result in the regrowth of TKIs‐sensitive clones and rapid tumor regrowth. In addition, the oligoprogressive disease might be treated with radiotherapy, whereas EGFR‐TKIs can be preserved and continued.^9^

In our study, patients treated with EGFR‐TKIs had a median PFS1 of 11.0 months, consistent with previous studies.^9^ Although it has been demonstrated that radiotherapy obtained good local control rates in NSCLC patients with oligometastatic disease at diagnosis,^9^, ^11^, ^12^, ^13^ data on the efficacy of local therapy such as radiotherapy on oligoprogressive disease after target therapy is scarce.^9^, ^14^ A recent study showed that local ablative therapy (radiation or surgery) and continuation of TKIs could extend disease control by >6 months in patients with *ALK*+ or *EGFR*‐MT NSCLC on erlotinib or crizotinib therapy who developed oligoprogressive disease.^9^ Consistent with this study, our results also demonstrated that radiotherapy with continued TKIs is effective for EGFR‐mutated NSCLC with oligoprogression.

Although radiotherapy may be effective for patients with oligoprogressive disease, the criteria for patient selection remain undetermined. In our study, the Cox regression model showed no significant correlation with PFS. The T790M mutation in exon 20 of the EGFR gene and MET amplification has been reported to be associated with EGFR‐TKIs resistance in NSCLC patients.^15^ Oligo‐clones, that are resistant to EGFR‐TKIs, may occur in oligoprogressive sites.^16^ Our study showed that the T790M mutation was common in patients with oligoprogression and was associated with better PFS. Although the results were not statistically significant, they could provide a theoretical basis for future research. Jing et al^17^ investigated the role of ionizing radiation in EGFR‐TKIs resistance caused by the T790M mutation in NSCLC cell lines. In this study, the H1975 is a cell line with EGFR double mutant (L858R plus T790M), while H3255 cell is an *EGFR* single mutant (L858R). The IC50 (gefitinib) of the 2.5 Gy group was statistically significant compared with that in the 0 Gy control group in H1975 cell lines (0.678 μmol/L vs. 3.520 μmol/L, *p* = 0.008). For H3255 cell line, no IC50 (gefitinib) difference was observed between the 2.5 Gy group and the 0 Gy control group (0.017 μmol/L vs. 0.041 μmol/L, *p* = 0.224). The above results indicated that ionizing radiation could reduce T790M mutation resistance in NSCLC cell lines.

Osimertinib is an oral and irreversible TKI selective for both EGFR T790M and EGFRm resistance mutations. Osimertinib is recommended for EGFR T790M‐positive NSCLC patients who have progressed on TKIs therapy. A study included 128 patients with T790M‐positive advanced NSCLC, who had progressed after prior TKIs treatment receiving osimertinib treatment, and the results demonstrated that median CNS PFS was not reached,^18^ indicating that osimertinib was a good choice for EGFR T790M‐positive NSCLC patients who failed TKIs treatment.

Our study had several limitations. Osoegawa A et al. demonstrated that frequent EGFR structural changes could occur after a long‐term history of EGFR‐TKIs therapy and a repeated tissue biopsy was useful in identifying the potential resistance mechanisms.^16^ Thus, it is recommended to make a rebiopsy to determine the genetic mutation status after TKIs resistance to guide further treatment. If tumor tissue was not available for re‐biopsy, a liquid biopsy could be performed. The sample size was relatively small and the present study was a retrospective study. In addition, our study lacked a comparator group and a consistent definition of oligoprogression. Despite these limitations, our study indicates that radiotherapy followed by continued treatment with EGFR‐TKIs is a safe and effective option for NSCLC patients with oligoprogression.

## 5 CONCLUSION

Radiotherapy with continued EGFR‐TKIs is an effective treatment for advanced NSCLC patients with oligoprogressive disease after first‐line EGFR‐TKIs. Multivariate analysis indicated that SBRT and PS of 0 to 1 were important prognostic factors for PFS2, and SBRT was an independent prognostic factor for OS. Moreover, patients with T790M mutation or radiotherapy within 1 month after oligometastasis had a better survival outcome. However, this difference was not statistically significant. Larger sample sizes and prospective and multicenter studies are needed to validate these clinical results in the future.

## AUTHORS’ CONTRIBUTIONS

Fang Wu designed the study. Chunhong Hu guided the study. Sixuan Wu selected patients and wrote the manuscript. Yuanqiang Wu and Renfang Deng assisted in manuscript revision. Yue Pan and Long Shu analyzed and interpreted the data. All authors approved the manuscript.

## FUNDING INFORMATION

Wu Jieping Foundation (320.6750.18535).

## CONFLICT OF INTEREST

The authors declare no conflicts of interest.

## Data Availability

Data supporting the results of this study are available upon request to the corresponding author.
